# Impact of a multidisciplinary care bundle for necrotizing skin and soft tissue infections: a retrospective cohort study

**DOI:** 10.1186/s13613-019-0598-4

**Published:** 2019-10-24

**Authors:** Tomas Urbina, Camille Hua, Emilie Sbidian, Romain Bosc, Françoise Tomberli, Raphael Lepeule, Jean-Winoc Decousser, Armand Mekontso Dessap, Olivier Chosidow, Nicolas de Prost, the Henri Mondor Hospital Necrotizing Fasciitis group

**Affiliations:** 1 Service de Réanimation Médicale, Hôpitaux Universitaires Henri Mondor, Assistance Publique-Hôpitaux de Paris (AP-HP), Créteil, France; 2 Service de Dermatologie, Hôpitaux Universitaires Henri Mondor, Assistance Publique-Hôpitaux de Paris (AP-HP), Créteil, France; 3 Université Paris-Est Créteil Val de Marne (UPEC), Créteil, France; 4 Epidemiology in Dermatology and Evaluation of Therapeutics (EpiDermE), EA7379, Université Paris Est-Créteil, DHU VIC, Créteil, France; 5 Service de Chirurgie Plastique et Reconstructrice, Hôpitaux Universitaires Henri Mondor, Assistance Publique-Hôpitaux de Paris (AP-HP), Créteil, France; 6 Service d’Anesthésie et des Réanimations Chirurgicales, Hôpitaux Universitaires Henri Mondor, Assistance Publique-Hôpitaux de Paris (AP-HP), Créteil, France; 7 Unité Transversale de Traitement des Infections, Hôpitaux Universitaires Henri Mondor, Assistance Publique-Hôpitaux de Paris (AP-HP), Créteil, France; 8 Laboratoire de Bactériologie-Hygiène, Hôpitaux Universitaires Henri Mondor, Assistance Publique-Hôpitaux de Paris (AP-HP), Créteil, France; 9 Équipe EA 7380 Dynamyc, Unité de Formation et de Recherche (UFR) de Médecine-Site Créteil, Université Paris-Est Créteil Val-de-Marne, Créteil, France; 10 Groupe de Recherche Clinique CARMAS, Université Paris Est-Créteil, Créteil, France

**Keywords:** Necrotizing skin and soft tissue infections, Necrotizing fasciitis, Patient care bundles, Multidisciplinary management, Time to debridement, Mortality

## Abstract

**Background:**

Necrotizing skin and soft tissue infections (NSTIs) require both prompt medical and surgical treatment. The coordination of multiple urgent interventions by care bundles has improved outcome in other settings. This study aimed to assess the impact of a multidisciplinary care bundle on management and outcome of patients with NSTIs.

**Methods:**

Patients with NSTIs admitted between 2006 and 2017 were compared according to admission before or after bundle implementation (2012–2013). This bundle consisted mainly in (1) the creation of a multidisciplinary task force; (2) management guidelines on empirical antibiotics, intensive care unit admission criteria, a triage algorithm to accelerate operating room access; and (3) an active communication policy. Patient recruitment and management were compared between pre- and post-implementation periods. Main outcome was day 60-censored hospital survival.

**Results:**

Overall, 224 patients were admitted: 60 before, 35 during, and 129 after bundle implementation. Admission after implementation was associated with increased yearly admissions (10 [8–13] vs 30 [24–43] patients/year, *p* = 0.014) and decreased mortality (30 vs 15%, HR = 0.49 [0.26–0.92]; *p* = 0.026) but was no longer a protective factor for mortality after adjustment on confounding factors (adjusted HR = 0.90 [0.43–1.88], *p* = 0.780). There was no significant difference regarding time to surgery (0 [0–1] vs 0 [0–1] days, *p* = 0.192) or rate of antibiotic treatment within 24 h (98% vs 99%, *p* > 0.99).

**Conclusions:**

Implementation of a multidisciplinary care bundle for NSTIs was feasible, but in a retrospective study from an already experienced center was not associated with significantly increased survival after adjustment.

## Background

Necrotizing skin and soft tissue infections (NSTIs) are rare, life-threatening bacterial infections resulting in extensive tissue necrosis and destruction. Hospital mortality ranges from 9.3 to 29.3%, with disabling sequelae for 30% of survivors [1–3]. With an incidence of 4/100,000 persons per year [4], initial misdiagnosis is frequent [5], leading to a delayed surgical debridement of infected tissues, one of the main modifiable prognostic factors [6]. Even when diagnostic issues have been overcome, further delays can occur because of logistical and technical difficulties regarding operating room emergency access [7]. Management in high case volume centers has been associated with enhanced survival [8], while a recent Cochrane review highlighted the lack of high quality evidence for a benefit on mortality for any single intervention [9]. This implies the need for a multimodal approach of NSTIs, as coordination of multiple urgent interventions has led to improved outcomes in other settings [10–13]. We hypothesized that standardizing and organizing early management at the hospital level would further improve patient care in our tertiary center, and implemented a multidisciplinary care bundle for patients with NSTIs. The current study aims at assessing the impact of this dedicated multimodal care protocol on the following endpoints: (1) yearly patient recruitment; (2) hospital mortality and secondary outcomes; and (3) early patient management, assessed with pre-defined endpoints.

## Methods

### Patients

We conducted a retrospective cohort study including all consecutive adult patients (≥ 18 years) admitted to our hospital for surgically confirmed NSTI from January 1st 2006 to December 31st 2017. Patients admitted between 2006 and 2013 were identified using the French national hospital database (Program for Medicalization of Information Systems) with the International Classification of Diseases diagnostic codes for “gangrene”, “infectious myositis”, “necrotizing fasciitis” and “cellulitis”. Patients admitted after 2014 were identified from a prospective NSTI database. Patients were excluded if NSTI was not surgically confirmed. Macroscopic appearance of tissues during operation (i.e., swollen, dull gray with a thin, brownish exudate with or without necrosis) was used as the main inclusion criterion as it is the gold standard for diagnosis of NSTI in the most recent guidelines [14] and no change within the surgical team occurred throughout the study period. Patients were also excluded if upon medical chart review NSTI was not the main admission diagnosis, or if there were missing data regarding the main outcome or two or more of the key management endpoints (i.e., time to surgery, time to antibiotics, adequacy of antibiotics to guidelines, admission to intensive care). Follow-up was conducted by review of electronic medical charts until end of study inclusion (December 31st 2017), using last available information (outpatient consultation or hospital discharge) to estimate follow-up time.

Patients received information during hospital stay that data abstracted from their medical charts could be used for research purposes. Data were anonymized and compiled according to the requirements of the *Commission Nationale Informatique et Liberté* (registration number 2003722) and the study was approved by the Comité de Protection des Personnes Ile-de-France V on March 8th 2018 (reference # 16165). The study has been reported according to the STROBE guidelines regarding observational cohort studies.

### NSTI care bundle

Through 2012 to 2013, a multidisciplinary bundle of care for NSTIs was progressively implemented in our tertiary referral center. It consisted in (1) the creation of a multidisciplinary task force involving intensive care physicians, dermatologists, surgeons, infectious diseases practitioners, microbiologists, and radiologists; (2) the use of a triage algorithm including a 24/7 on-call dermatologist for patient referral and a multidisciplinary bedside assessment to facilitate access to the operating room; (3) the implementation of local management guidelines addressing empiric antibiotic treatment, intensive care unit (ICU) admission criteria, prioritization for operating room access, adequate specimen collection for laboratory detection of responsible microorganisms, systematic “second-look” surgery recommendation 24 h after initial surgical debridement, together with a routine multidisciplinary bedside reassessment during the post-operative period; (4) the prospective identification of all NSTI cases admitted to our institution as well as their inclusion in a dedicated database; (5) trimesterly review of all NSTI cases by the multidisciplinary task force; and (6) the conduction of research projects and an active communication policy towards the medical community about the existing bundle. The main elements of this bundle are presented in Additional file 1: Figures S1 and S2 and Additional file 2: Appendix S1.

### Study design

Using a before–after design, we compared patients from the pre- and post-implementation period (2006–2011 vs 2014–2017) for the following variables: number of yearly admissions, patients’ clinical characteristics, key pre-defined early management endpoints (i.e., time from hospital admission to first surgical debridement (measured in days), antibiotic administration within 24 h of hospital admission, adequacy of antibiotics to guidelines, ICU admission), number of surgical debridements, length of hospital stay and hospital mortality. Shock was defined as need for vasopressors, amputation was defined as amputation of at least a limb segment, of external genitalia or of perineal sphincters. Initial symptoms and their time of onset were recovered from medical charts or considered as missing if not reported. Microbiological data were obtained from samples collected during the first surgery, blood cultures, subcutaneous and bullae punctures collected before or on the day of the first surgery. Samples obtained from subsequent surgical procedures were not included. Results from all samples were merged to categorize infections as mono- or polymicrobial for each patient. All data were collected upon medical chart review. Due to the progressive implementation of the different bundle items, patients admitted between January 2012 and December 2013, the defined implementation period, were excluded from the final analysis. The primary endpoint was 60-day-censored hospital survival. Primary outcome and key management outcomes had been defined a priori. The adequacy of empirical antibiotic treatment was defined according to the most recent French [15] and international guidelines [1–3].

### Statistical analysis

Continuous variables were reported as median [1st–3rd quartiles] and categorical data as percentages. No imputation was performed for missing data, except for comorbidities, imputed as absent if not otherwise stated. Differences between patients included during the pre- and post-implementation periods were tested using the Mann–Whitney non-parametric test for continuous variables, and the Fisher’s exact test or the Chi-squared test for categorical variables, according to sample size. A sensitivity analysis for the impact of bundle implementation on pre-defined management endpoints was conducted including only patients presenting with shock. Factors associated with day 60-censored hospital survival were identified using uni- and multivariable Cox proportional hazards regression models, hazard ratios (HR) and their 95% confidence interval (CI) were computed. Features yielding a *p* value < 0.05 in univariable analysis were included in the multivariable model, with a manual backwards stepwise elimination procedure of variables displaying a *p* value greater than 0.10 until reaching the final model. No imputation was made and patients with missing data for one of the variables from the model were excluded from the analysis. Sensitivity analyses were conducted applying the same model to either the whole study population, including patients from the implementation period, or to only patients from the pre- and post-implementation periods presenting with shock. Survival curves were drawn using the Kaplan–Meier survival analysis, and the log rank test was used to compare the differences. Significance was defined as two-sided *p* value < 0.05. Data were collected and entered into a Microsoft Excel 2010 (Microsoft Corporation, Redmond, WA) spreadsheet. All statistical analyses were performed using the R software (v 2.12.0; http://cran.r-project.org). Tables and figures were made using R and GraphPad Prism 5 (GraphPad Software Inc, La Jolla, Calif).

## Results

During the study period (2006–2017), 282 patients were admitted with a diagnosis of NSTI, including 44 patients for whom surgically confirmed NSTI was not the main admission diagnosis (no surgical confirmation, *n* = 37, and hospital admission for another reason than NSTI care, *n* = 7) and 14 patients with missing data [regarding main outcome (*n* = 10), regarding two or more predefined key management endpoints (*n* = 4)], leaving 224 patients available for study inclusion. Of these, 35 patients were admitted during the implementation period, resulting in 189 patients available for analysis (60 from the pre-implementation period and 129 from the post-implementation period) (see Additional file 1: Figure S3). The median follow-up time was 86 [30–367] days.

### Impact of the NSTI care bundle on patient recruitment and characteristics

**Fig. 1:**
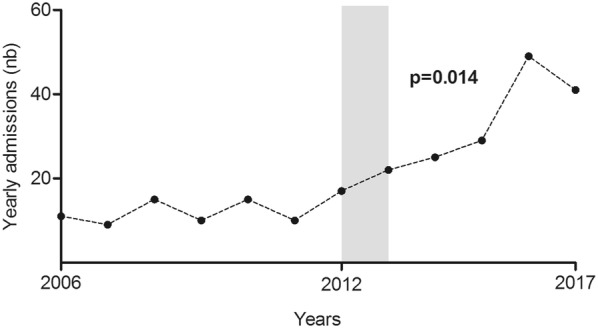
Yearly admissions to our center for necrotizing soft tissue infections (dotted line). The grayed area represents the implementation period of our bundle of care. *p* value for a Mann–Whitney test comparing yearly admissions for NSTI between the pre- and post-implementation periods

Between the pre- and post-implementation periods, there was a marked increase in the yearly number of patients admitted to our center for NSTIs (10 [8–13] vs 30 [24–43] patients/year, *p* = 0.014; Fig. 1), with no increase in the proportion of patients transferred from another center (52% vs 53%, *p* = 0.939). Regarding patients’ characteristics upon admission, the only significant difference was a lower rate of nosocomial NSTIs during the post-implementation period (33% vs 13%, *p* = 0.002) and a trend for NSTIs to more frequently affect the lower limbs (67% vs 80%, *p* = 0.060). There were no significant differences between the two periods regarding non-steroidal anti-inflammatory drug and antibiotic use before hospital admission, the presence of shock upon admission (Table 1), or the microbiological documentation of NSTIs (see Additional file 1: Table S1).

**Table 1:** Demographics, comorbidities and clinical features upon admission of patients with necrotizing skin and soft tissue infections before (*n* = 60), during (*n* = 35) and after (*n* = 129) the implementation of a dedicated multimodal and multidisciplinary bundle

	Available data	All patients *n* = 224	Pre-implementation period (2006–2011) *n* = 60	Implementation period (2012–2013) *n* = 35	Post-implementation period (2014–2017) *n* = 129	*p* ^a^
Demographics						
Age, years, median (IQR)	224	64 (53–74)	66 [55–76]	68 [60–75]	61 [53–72]	0.193
Male gender, *n* (%)		127 (56.7)	34 (56.7)	17 (48.6)	76 (58.9)	0.894
Comorbidities, *n* (%)						
Diabetes mellitus	224	83 (37.1)	18 (30.0)	12 (34.3)	53 (41.1)	0.192
Immunodeficiency		58 (25.9)	13 (21.7)	14 (40.0)	31 (24.0)	0.863
HIV infection		2 (0.9)	1 (1.7)	1 (2.9)	0 (0.0)	0.694
Cancer		21 (9.4)	5 (8.3)	7 (20.0)	9 (7.0)	0.974
Corticosteroids		36 (16.1)	6 (10.0)	9 (25.7)	21 (16.3)	0.355
Obliterating arteritis of the lower limbs		24 (10.7)	6 (10.0)	4 (11.4)	14 (10.9)	> 0.99
Liver cirrhosis		9 (4.0)	1 (1.7)	2 (5.7)	6 (4.7)	0.550
Chronic kidney disease		25 (11.2)	7 (11.7)	5 (14.3)	13 (10.1)	0.939
Chronic alcohol consumption		27 (12.1)	7 (11.7)	5 (14.3)	15 (11.6)	> 0.99
Obesity		57 (25.4)	16 (26.7)	10 (28.6)	31 (24.0)	0.834
Prior to admission						
Time from first symptom, days, median (IQR)	214	5 [2–10]	6 [2–12]	4 [1–15]	5 [3–9]	0.848
Antibiotic treatment, *n* (%)	221	137 (61.2)	33 (55.9)	20 (58.8)	84 (65.6)	0.267
NSAID use, *n* (%)	222	46 (20.5)	9 (15.0)	4 (11.8)	33 (26.0)	0.143
Transferred from another center, *n* (%)	223	116 (51.8)	31 (51.7)	16 (47.1)	69 (53.5)	0.939
Presentation upon admission						
Nosocomial infection, *n* (%)	222	45 (20.1)	20 (33.3)	8 (23.5)	17 (13.4)	*0.002*
Infection site, *n* (%)	223					
Inferior limbs		173 (77.2)	40 (66.7)	30 (85.7)	103 (80.5)	0.060
Superior limbs		19 (8.5)	8 (13.3)	1 (2.9)	10 (7.8)	0.351
Abdomino-perineal infection		38 (17.0)	13 (21.7)	4 (11.4)	21 (16.4)	0.503
Cervico-facial infection		2 (0.9)	0 (0.0)	0 (0.0)	2 (1.6)	0.833
Other		1 (0.4)	0 (0.0)	0 (0.0)	1 (0.8)	1
Multifocal infection, *n* (%)	223	23 (10.3)	5 (8.3)	4 (11.4)	14 (10.9)	0.770
Shock, *n* (%)	220	91 (40.6)	30 (50.8)	12 (34.3)	49 (38.9)	0.170

*HIV* human immunodeficiency virus, *NSAID* non-steroidal anti-inflammatory drug

^a^*p* values for univariate comparison of the pre- and post-implementation periods; Chi-squared test or Fisher’s exact test were used for categorical data according to sample size, Mann–Whitney’s test were used for continuous variables due to non-parametrical distribution

### Impact of the NSTI care bundle on day 60-censored hospital survival and secondary outcomes

**Fig. 2:**
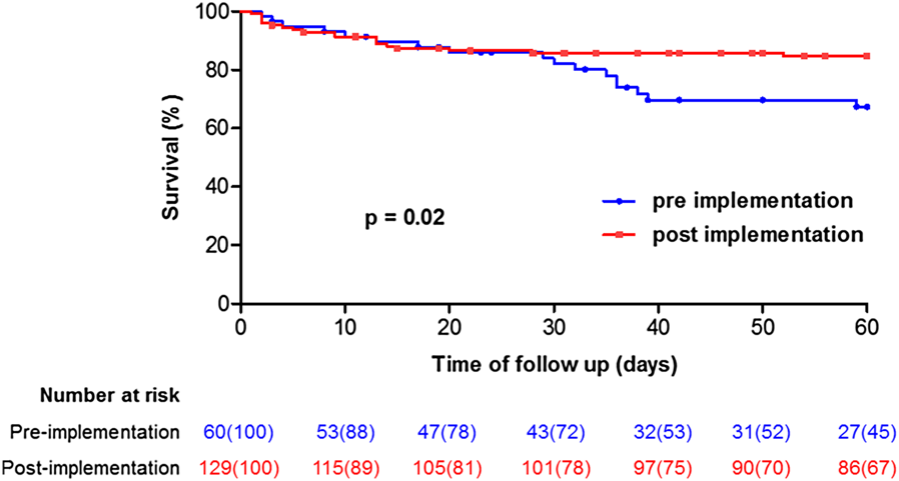
Kaplan–Meier survival curves comparing patients admitted during the pre- (blue line) and post- (red line) bundle of care implementation periods. *p* value comes from an unadjusted log rank test. Survival was censored at 60 days

Overall, 51 patients (23%) had died 60 days after admission, with a significantly lower mortality after bundle implementation (30% (*n* = 18/60) vs 15% (*n* = 20/129), *p* = 0.034). Length of hospital stay was shorter (29 [17–41] vs 21 [11–34] days, *p* = 0.041), with no difference in the rate of amputation (22% vs 16%, *p* = 0.418) or the median number of debridements (1 [1–2] vs 1 [1–2], *p* = 0.587). Consistently, admission during the post-implementation period was a protective factor for day 60-censored hospital survival in univariable analysis (HR = 0.49 [0.26–0.92]; *p* = 0.026) (Fig. 2). All admission characteristics associated with mortality in univariable analysis are presented in Table 2. In a multivariable Cox model, the variables remaining associated with mortality were age as a continuous variable (adjusted (a) HR = 1.04 [1.01–1.07], *p* = 0.011), immunodeficiency (aHR = 2.20 [1.09–4.44], *p* = 0.028), the nosocomial status of the infection (aHR = 2.28 [1.00–5.16], *p* = 0.049) and the presence of shock (aHR = 8.13 [3.26–20.20], *p* < 0.001). Antibiotics administered before admission were a protective factor (aHR = 0.36 [0.17–0.75], *p* = 0.006). The admission period was no longer associated with day 60-censored hospital survival (aHR = 0.90 [0.43–1.88], *p* = 0.780) (Table 2). Two sensitivity analyses including either all cohort patients, even those from the implementation period (2011–2012), or only those presenting with shock upon admission, yielded consistent results (see Additional file 1: Tables S2 and S3). The main management endpoints identified a priori (time to first surgery, rate of patients undergoing surgery in the first 24 h, rate of patients receiving antibiotics in the first 24 h and rate of adequacy of first antibiotherapy to guidelines) were not significantly associated with survival (see Additional file 1: Table S4).

**Table 2:** Admission characteristics associated with hospital mortality censored at day 60 amongst patients admitted in the pre- and post-implementation periods

	Available data	Survivors *n* = 151	Non-survivors *n* = 38	Unadjusted hazard ratio [95% CI]^a^	*p* ^b^	Adjusted hazard ratio [95% CI]^c^	*p* ^d^
Inclusion period, *n* (%)							
Post-implementation	129	109 (72.2)	20 (52.6)	0.49 [0.26–0.92]	*0.026*	0.90 [0.43–1.88]	0.780
Pre-implementation	60	42 (27.8)	18 (47.4)	**–**	**–**		
Demographical data							
Age, years, median (IQR)	189	61 [51–72]	71 [59–80]	1.00 [1.00–1.10]	*0.018*	1.04 [1.01–1.07]	*0.011*
Male gender, *n* (%)		86 (57.0)	24 (63.2)	–	0.510		
Comorbidities, *n* (%)							
Diabetes mellitus	189	58 (38.4)	13 (34.2)	–	0.520		
Immunodeficiency		26 (17.2)	18 (47.4)	3.30 [1.70–6.20]	*< 0.001*	2.20 [1.09–4.44]	*0.028*
HIV infection		1 (0.7)	0 (0.0)	–	> 0.99		
Cancer		9 (6.0)	5 (13.2)	–	0.140		
Corticosteroids		16 (10.6)	11 (28.9)	2.70 [1.40–5.50]	*0.005*		
Obliterating arteritis of the lower limbs		15 (9.9)	5 (13.2)	–	0.580		
Liver cirrhosis		4 (2.6)	3 (7.9)	–	0.100		
Chronic kidney disease		10 (6.6)	10 (26.3)	3.50 [1.70–7.20]	*< 0.001*	**–**	**–**
Chronic alcohol consumption		20 (13.2)	2 (5.3)	–	0.190		
Obesity		42 (27.8)	5 (13.2)	–	0.061		
Prior to admission							
Time from first symptom, days, median (IQR)	180	5 [3–9]	3 [1–8]	–	0.410		
Antibiotic treatment, *n* (%)	187	99 (65.6)	18 (47.4)	0.51 [0.27–0.97]	*0.041*	0.36 [0.17–0.75]	*0.006*
NSAID use, *n* (%)	188	38 (25.2)	4 (10.5)	–	0.076		
Transferred from another center, *n* (%)	189	79 (52.3)	21 (55.3)	–	0.670		
Presentation upon admission							
Nosocomial infection, *n* (%)	188	24 (15.9)	13 (34.2)	2.50 [1.30–5.00]	*0.007*	2.28 [1.00–5.16]	*0.049*
Infection site, *n* (%)	188						
Inferior limbs		114 (75.5)	29 (76.3)	–	0.600		
Superior limbs		15 (9.9)	3 (7.9)	–	0.690		
Abdomino-perineal		27 (17.9)	7 (18.4)	–	0.960		
Cervico-facial		2 (1.3)	0 (0.0)	–	> 0.99		
Other		1 (0.7)	0 (0.0)	–	> 0.99		
Multifocal infection, *n* (%)	188	14 (9.3)	5 (13.2)	–	0.540		
Shock, *n* (%)	185	48 (31.8)	31 (81.6)	8.20 [3.40–20.00]	*< 0.001*	8.13 [3.26–20.20]	*< 0.001*

*HIV* human immunodeficiency virus, *NSAID* non-steroidal anti-inflammatory drugs

Univariable and multivariable analysis by Cox proportional hazards model. Multivariable analysis for 179 patients (10 excluded for missing data on one of the variables)

^a,b^Hazard ratios and *p* values from the comparison of survivors and non-survivors by univariate cox regression analysis for survival censored at 60 days

^c,d^Adjusted hazard ratios and *p* values from a multivariate cox model for survival censored at 60 days. Only patients from the pre- and post-implementation periods were included in the analysis. Variables included in the model were all variables available upon admission associated with mortality in the univariate model with a *p* value inferior or equal to 0.05. Corticosteroid treatment was included as part of immunodeficiency. Included variables with a *p* value > 0.1 (chronic kidney disease) in the multivariate model were excluded from the final model

### Impact of the NSTI care bundle on patient management

**Fig. 3:**
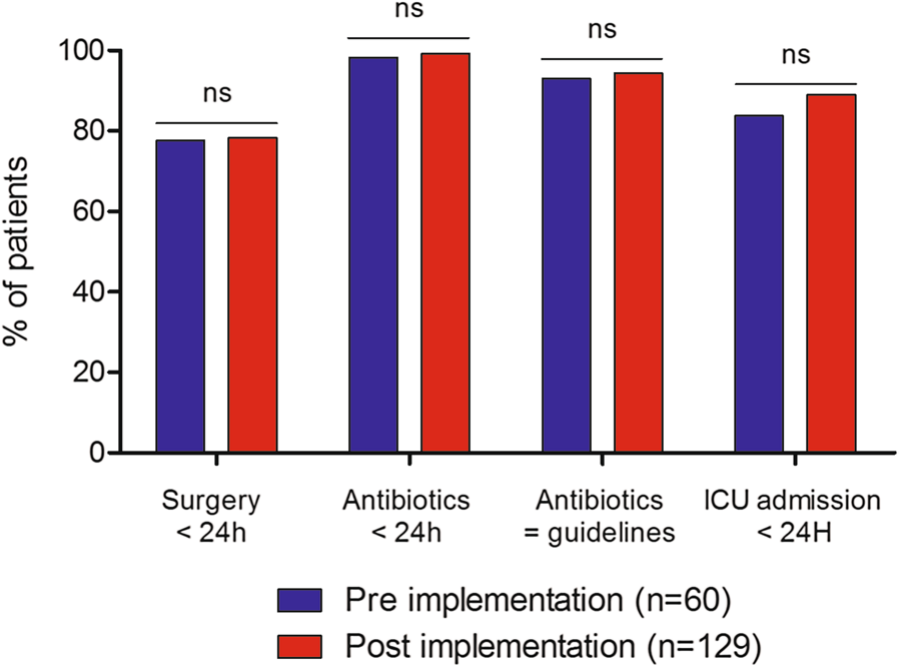
Impact of the implementation of a multimodal bundle of care on pre-defined patient management outcomes. Patients from the pre- (blue histograms) and post-implementation (red histograms) periods are compared. None of the comparisons yielded statistically different results by Fisher’s exact test or the Chi-squared test according to sample size

No significant differences in key pre-defined management endpoints were noted between the pre- and post-implementation periods (Fig. 3). The time to first surgery was 0 [0–1] vs 0 [0–1] days (*p* = 0.192), with 78% of patients undergoing surgery within the first 24 h in both groups. Antibiotics were administered within the first 24 h in more than 98% of patients in both groups, and in accordance to guidelines in more than 90% of cases. The rate of ICU admission did not significantly differ between the two periods, nor did the rate of ICU admission within the first 24 h (see Additional file 1: Table S5). A sensitivity analysis including the most severe patients, i.e., those presenting with shock, did not reveal any significant differences for these same endpoints (see Additional file 1: Table S6). Of 224 patients, seven received intravenous immunoglobulins: one from the implementation period and six from the post-implementation period. Hyperbaric oxygen therapy was not used.

## Discussion

The main results of this retrospective study assessing the impact of the implementation of a multidisciplinary NSTI care bundle are as follows: (1) the NSTI care bundle was associated with a marked increase in yearly admissions; (2) it was associated with an increase in day 60-censored hospital survival in univariable analysis, which was not maintained after adjustment for admission characteristics; and (3) it did not allow for significantly altering key pre-defined patient management endpoints.

The increase in yearly patient recruitment (Fig. 1) per se could be an interesting benefit of our bundle. Indeed, in a recent study by Audureau et al. [8], patients managed in centers admitting a high volume of NSTI cases (defined by 3 or more yearly cases) had a lower 28-day mortality, even after adjusting for potential confounding factors. Bernal et al. found that mortality was lower for patients whose initial surgical debridement was performed by a surgeon experienced in NSTIs [16], further highlighting the potential benefits of being managed in centers with high case volumes. Although yearly patient recruitment increased between the pre- and post-implementation periods, there was no increase in the rate of patients who were transferred to our center from another facility. As the incidence of NSTI is not thought to be rising [17, 18], this could nevertheless be due to our communication policy, by an increase of direct referral by pre-hospital medical teams or general practitioners as well as by an increase of spontaneous patient consultation to our center’s  “dermatological emergency ward”. As the inclusion criteria for our study was surgically confirmed NSTI, an increase in incidence due to erroneous diagnosis is unlikely, but could be due to the increased awareness, experience and training of physicians regarding NSTI in our center.

The survival benefit associated with the post-implementation period was not maintained after adjustment for admission characteristics (Fig. 2, Table 2). The main hypothesis is that bundle impact could have been undermined because our center was already experienced in the management of NSTIs. Indeed, there was no significant difference regarding the main management endpoints between the pre- and post-implementation periods (Fig. 3). The rate of patients rapidly undergoing surgery and receiving adequate antibiotics, even before implementation of the bundle, is difficult to compare to the literature. It was markedly high compared to some studies [19], and lower compared to others [20, 21]. Nevertheless, the rate of patients transferred from other centers in our series, of more than 50% both in the pre- and post-implementation periods, is much higher than in previous studies evaluating NSTIs at a national level (Audureau et al. 13% [8], Holena et al. 10% [22], Ingraham et al. 30% [23]) but similar to that of series from experienced centers such as the one by Bernal et al. (59%) [16]. Besides this homogenous management, other explaining factors could be a lack of power due to the rarity of NSTIs or a change in patient characteristics associated with the increase in the number of admissions, as highlighted by the lesser proportion of patients with nosocomial infections, known to be more severe [24, 25]. Figure 1 illustrates that the mortality difference between groups seems to develop after day 30. We speculate this could reflect the impact of age and pre-existing comorbidities on mortality, rather than that of the first 48 h of management on which our bundle mainly focused. Finally, the fact that antibiotics administered before admission were a protective factor for hospital survival in the multivariable model is remarkable and may have limited our ability to demonstrate a benefit of antibiotics administered within 24 h of hospital admission.

This work has several limitations, the first of which being its monocentric retrospective design, limiting the generalization of its results. Second, time to surgery was measured in days, not in hours, which could have undermined our ability to demonstrate its impact on outcome. As patients were identified with two different methods according to the period of inclusion (electronic records or prospective database) and because 14 patients with important missing data were excluded without imputation, we cannot exclude a selection bias. Antibiotic treatment was not evaluated for adequacy to documentation, duration, de-escalation and side effects. We could not obtain time to administration in hours, due to the retrospective design of the study. Finally, elements not included in our bundle and that were not evaluated could have impacted outcome, such as use of anti-toxinic antibiotics or negative-pressure wound therapy [26].

Our study also has several strengths, the first of which is a well-defined diagnosis as a main inclusion criterion. Indeed, an important part of the literature on NSTIs has included patients based on an electronic record diagnosis of NSTIs, a major selection bias for a disease with a challenging diagnosis [23]. We only included surgically proven NSTIs, and in spite of this restrictive definition, the second strength of this cohort is its large size. Finally, we applied a rigorous methodology to this before–after study, defining key management endpoints a priori, and choosing a strong outcome measure (i.e., hospital mortality).

Interestingly, during the conduction of this project, the only other report, to our knowledge, of a multidisciplinary bundle of care for NSTIs was published. Although focusing on ICU patients, this much smaller series found a very similar benefit on mortality to their bundle (40% vs 15% after implementation, compared to 30% vs 15% in our work), highlighting this approach’s potential interest [27]. By contrast to the hospital that conducted this research, our center has been a referral center for NSTIs for several decades. Standard of care before bundle implementation likely rendered patient management homogeneous, making it difficult to show a statistically significant difference on a solid outcome like mortality. Nevertheless, our work confirms the feasibility of standardizing multidisciplinary care for NSTIs on a larger scale.

## Conclusions

Although this retrospective work from a center with preexisting expertise could not find a benefit on mortality to the implementation of a multidisciplinary care bundle for NSTIs after adjustment for confounding factors, it highlights its feasibility and potential interests, such as increasing patient recruitment. Every practitioner confronted with this rare, severe and often little-known condition, especially at the time of triage, could benefit from implementing a multidisciplinary bundle appointing specialists involved in NSTI management to optimize and speed up treatment. Future work, ideally prospective, could benefit from and abound in favor of the approach we describe.

## Supplementary information

**Additional file 1.** The study was approved by the Comité de Protection des Personnes Ile-de-France V on March 8th 2018 (reference # 16165).

**Additional file 2: Appendix S1.** Details on the necrotizing soft-tissue infection task force and on bundle items.

## Data Availability

The datasets used and/or analyzed during the current study are available from the corresponding author on reasonable request.

## Abbreviations

aHR: adjusted hazard ratio
CI: confidence interval
HIV: human immunodeficiency virus
HR: hazard ratio
ICU: intensive care unit
IQR: interquartile range
NSAID: non-steroidal anti-inflammatory drug
NSTI: necrotizing soft tissue infection
