# Supplementary material for: Impact of a multidisciplinary care bundle for necrotizing skin and soft tissue infections: a retrospective cohort study
Source: Ann Intensive Care. 2019 Oct 24;9:123. doi: 10.1186/s13613-019-0598-4 (PMC6813408; doi:10.1186/s13613-019-0598-4)
Supplement: Supplementary file 1 — Additional file 1. The study was approved by the Comité de Protection des Personnes Ile-de-France V on March 8th 2018 (reference # 16165). [file 13613_2019_598_MOESM1_ESM.docx]

**Impact of a multidisciplinary care bundle for necrotizing skin and soft tissue infections: a retrospective cohort study**

**Additional File 1: Tables S1 to S5 and Figures S1 to S3**

**Table S1. Microbiological data** for all 224 patients admitted with necrotizing soft tissue infection.

|  | **All patients**  **n= 224** | **Pre-implementation**  **Period (2006-2011),**  **n= 60** | **Implementation period (2012-2013), n=35** | **Post-implementation**  **Period (2014-2017), n= 129** | **p^a^** |
| --- | --- | --- | --- | --- | --- |
| No microbial documentation | 18 (8.0) | 7 (11.7) | 1 (2.9) | 10 (7.8) | 0.547 |
| Monomicrobial infection | 93 (41.5) | 19 (35.8) | 17 (50.0) | 57 (47.9) | 0.193 |
| Polymicrobial infection | 113 (50.5) | 34 (64.2) | 17 (50.0) | 62 (52.1) | 0.193 |
| *Staphylococcus spp.* | 80 (35.7) | 21 (35.0) | 10 (28.6) | 49 (38.0) | 0.815 |
| *Streptococcus spp.* | 101 (45.1) | 24 (40.0) | 19 (54.3) | 58 (45.0) | 0.629 |
| *Enterobacteriaceae* | 80 (35.7) | 23 (38.3) | 15 (42.9) | 42 (32.6) | 0.540 |
| Anaerobic bacteria | 23 (10.3) | 6 (10.0) | 3 (8.6) | 14 (10.9) | >0.99 |
| Non-fermenting gram-negative bacilli | 49 (21.9) | 9 (15.0) | 9 (25.7) | 31 (24.0) | 0.221 |
| Others | 42 (18.8) | 11 (18.3) | 5 (14.3) | 26 (20.2) | 0.923 |
| Bacteraemia | 46 (20.5) | 14 (23.3) | 4 (11.4) | 29 (22.5) | >0.99 |

^a^ p values for univariate comparison of the pre and post-implementation periods; Chi-squared test or Fisher’s exact test were used for categorical data according to sample size.

**Table S2. Sensitivity analysis for the multivariate Cox model assessing the relationship between the inclusion period and hospital mortality** (censored at day 60). **Model including patients from every inclusion period**: pre-implementation period was compared to per- and post implementation periods (n=213. 11 patients excluded for missing data on one or more of the variables).

|  | **Unadjusted Hazard Ratio  [95% CI]^a^** | **P^b^** | **Adjusted Hazard Ratio  [95% CI]^c^** | **p^d^** |
| --- | --- | --- | --- | --- |
| Post-implementation period | 0.44 [0.25-0.78] | 0.005 | 0.68 [0.36-1.28] | 0.235 |
| Age | 1.00 [1.00-1.00] | 0.011 | 1.03 [1.01-1.05] | 0.015 |
| Immunodeficiency | 2.60 [1.50-4.40] | <0.001 | 1.56 [0.84-2.88] | 0.157 |
| Nosocomial infection | 2.90 [1.70-5.20] | <0.001 | 3.24 [1.73-6.04] | <0.001 |
| Antibiotic treatment before admission | 0.53 [0.30-0.92] | 0.024 | 0.44 [0.24-0.81] | 0.008 |
| Shock upon admission | 5.5 [2.9-11] | <0.001 | 4.91 [2.54-9.50] | <0.001 |

Variables included in the model were all variables from the model used in Table 4, available upon admission and associated with mortality in the univariate model from Table 3 with a p value inferior or equal to 0.05. Corticosteroid treatment was included as part of immunodeficiency. Included variables with a p value > 0.1 (chronic kidney disease) in the multivariate model were excluded from the final model.
^a.b^ Hazard ratios and p values from the comparison of survivors and non-survivors by univariate cox regression analysis.
^c.d^ Adjusted hazard ratios and p values from a multivariate cox model for survival.

**Table S3**. **Sensitivity analysis for the multivariate Cox model assessing the relationship between the inclusion period and hospital mortality** (censored at day 60). **Model including only patients presenting with shock upon admission from the pre and post-implementation periods** (n=76, 3 patients excluded for missing data on one or more of the variables).

|  | **Unadjusted Hazard Ratio  [95% CI]^a^** | **P^b^** | **Adjusted Hazard Ratio  [95% CI]^c^** | **p^d^** |
| --- | --- | --- | --- | --- |
| Post-implementation period | 0,57 [0,28-1,20] | 0,37 | 0,90 [0,40-2,05] | 0,8073 |
| Age | 1,00 [0,99-1,10] | 0,120 | 1,03 [1,00-1,06] | 0,084 |
| Immunodeficiency | 2,40 [1,20-4,80] | 0,017 | 2,06 [0,95-4,49] | 0,068 |
| Nosocomial infection | 2,20 [1,00-4,60] | 0,040 | 2,39 [0,96-5,94] | 0,062 |
| Antibiotic treatment before admission | 0,61 [0,30-1,20] | 0,170 | 0,35 [0,15-0,82] | 0,016 |

Variables included in the model were all variables from the model used in Table 4, available upon admission and associated with mortality in the univariate model of Table 3 with a p value inferior or equal to 0.05. Corticosteroid treatment was included as part of immunodeficiency. Included variables with a p value > 0.1 (chronic kidney disease) in the multivariate model were excluded from the final model.
^a.b^ Hazard ratios and p values from the comparison of survivors and non-survivors by univariate cox regression analysis.
^c.d^ Adjusted hazard ratios and p values from a multivariate cox model for survival.

**Table S4. Univariable Cox proportional hazards model: association of management endpoints with hospital mortality** censored at day 60, amongst patients admitted in the pre- and post-implementation periods.

|  | **Available data** | **Survivors**  **n= 151** | **Non-survivors**  **n= 38** | **Unadjusted hazard ratio [95% CI]^a^** | **p^b^** |
| --- | --- | --- | --- | --- | --- |
| **Management** | | | | | |
| Time to first surgery, days, median (IQR) | 187 | 0 [0-1] | 0 [0-1] | 0.96 [0.84-1.1] | 0.560 |
| Surgery in the first 24 hours. n (%) | 187 | 117 (77.5) | 29 (76.3) | 1.1 [0.5-2.4] | 0.820 |
| Antibiotics in the first 24 hours. n (%) | 187 | 148 (98.0) | 37 (97.4) | 0.35 [0.05-2.40] | 0.300 |
| Antibiotics adequate to guidelines. n (%) | 186 | 137 (90.7) | 36 (94.7) | 2.8 [0 .39-21) | 0.300 |
| Intensive care unit admission <24h | 110 | 70 (89.7) | 29 (90.6) | - | >0.99 |

^a.b^ Hazard ratios and p values from the comparison of survivors and non-survivors by univariate cox regression analysis. Survival censored at 60 days. Intensive care unit admission < 24h evaluated only in the intensive care unit admitted population with an available admission delay (n=110. survivors n=78. non survivors=32).

**Table S5. Management of patients admitted for necrotizing skin and soft tissue infections** **before (n=60), during (n=35) and after (n=129) the implementation of a dedicated multimodal and multidisciplinary bundle**.

|  | **Available data** | **All patients**  **n= 224** | **Pre-implementation**  **Period (2006-2011)**  **n= 60** | **Implementation period (2012-2013), n=35** | **Post-implementation**  **Period (2014-2017), n= 129** | **p^a^** |
| --- | --- | --- | --- | --- | --- | --- |
| **Pre-defined management endpoints** | | | | | | |
| Time to first surgery, days, median (IQR) | 222 | 0 [0-1] | 0 [0-1] | 0 [0-2] | 0 [0-1] | 0.192 |
| Surgery in the first 24 hours, n (%) | 222 | 170 (75.9) | 45 (77.6) | 24 (68.6) | 101 (78.3) | >0.99 |
| Antibiotics in the first 24 hours, n (%) | 221 | 217 (96.9) | 57 (98.3) | 32 (94.1) | 128 (99.2) | >0.99 |
| Antibiotics adequate to guidelines, n (%) | 219 | 205 (91.5) | 53 (93.0) | 32 (97.0) | 119 (94.4) | >0.99 |
| Intensive care unit admission, n (%) | 224 | 134 (59.8) | 39 (65) | 22 (62.9) | 73 (56.6) | 0.349 |
| If yes, <24h, n (%) | 131 | 113 (86.3) | 31 (83.8) | 17 (77.2) | 65 (89.0) | 0.546 |

^a^ p values for univariate comparison of the pre and post-implementation periods; Chi-squared test or Fisher’s exact test were used for categorical data according to sample size, Mann-Whitney’s test were used for continuous variables due to non-parametrical distribution.

**Table S6. Subgroup analysis of shocked patients for management of patients admitted for necrotizing skin and soft tissue infections before (n=30), and after (n=49) the implementation of a dedicated multimodal and multidisciplinary bundle.**

|  | **Available data** | **All patients**  **n= 79** | **Pre-implementation**  **Period (2006-2011)**  **n= 30** | **Post-implementation**  **Period (2014-2017), n= 49** | **p^a^** |
| --- | --- | --- | --- | --- | --- |
| **Management** | | | | | |
| Time to first surgery, days, median (IQR) | 78 | 0 [0-0] | 0 [0-1] | 0 [0-0] | 0.136 |
| Surgery in the first 24 hours, n (%) | 78 | 66 (84.6) | 23 (79.3) | 43 (87.8) | 0.500 |
| Antibiotics in the first 24 hours, n (%) | 79 | 78 (98.7) | 29 (96.7) | 49 (100) | 0.830 |
| Antibiotics adequate to guidelines, n (%) | 78 | 76 (97.4) | 28 (96.6) | 48 (98.0) | >0.99 |
| Intensive care unit admission, n (%) | 79 | 78 (98.7) | 29 (96.7) | 49 (100) | 0.803 |
| If yes, <24h, n (%) | 77 | 67 (87) | 23 (82.1) | 44 (89.8) | 0.543 |

^a^ p values for univariate comparison of the pre and post-implementation periods; Chi-squared test or Fisher’s exact test were used for categorical data according to sample size, Mann-Whitney’s test were used for continuous variables due to non-parametrical distribution.

**Figure S1. Main components of the multidisciplinary and multimodal bundle implemented in our center for the care of patients with necrotizing soft tissue infections.** The multidisciplinary team is composed of dermatologists. intensive care and infectious disease practitioners. surgeons and microbiologists.


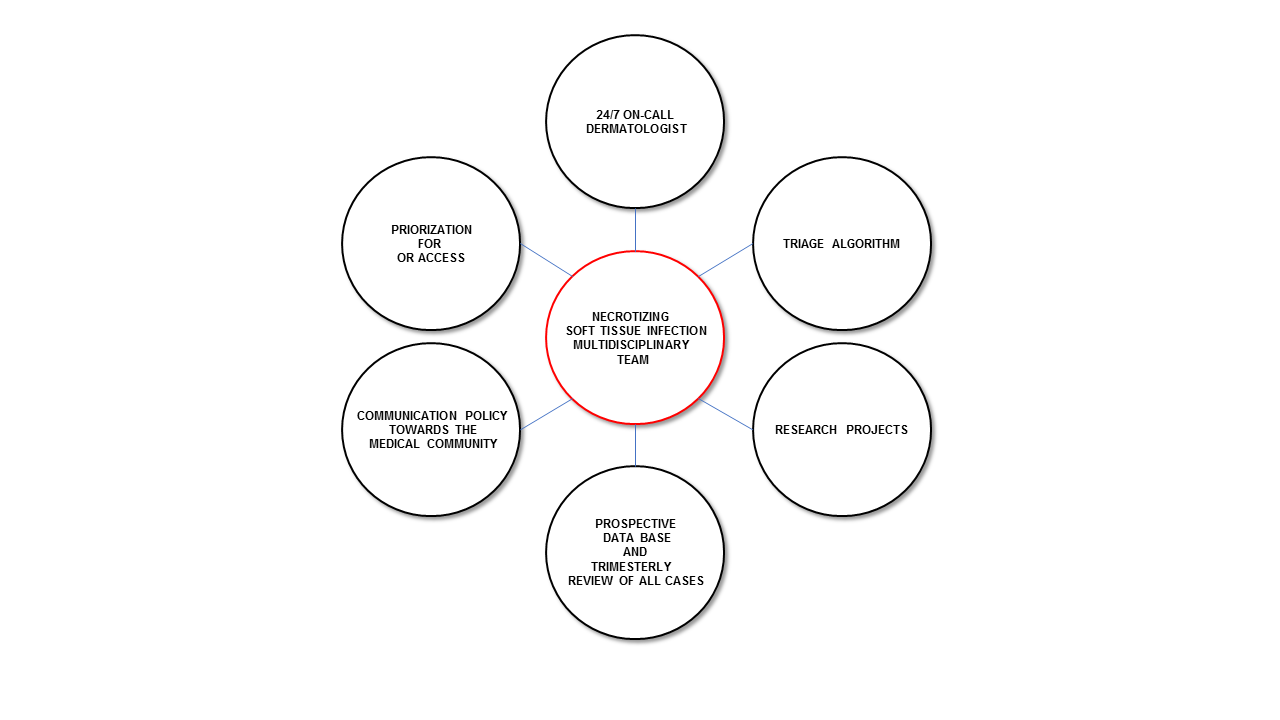


**Figure S2**. **Triage algorithm for necrotizing soft tissue infections in our center**. ICU: intensive care unit.
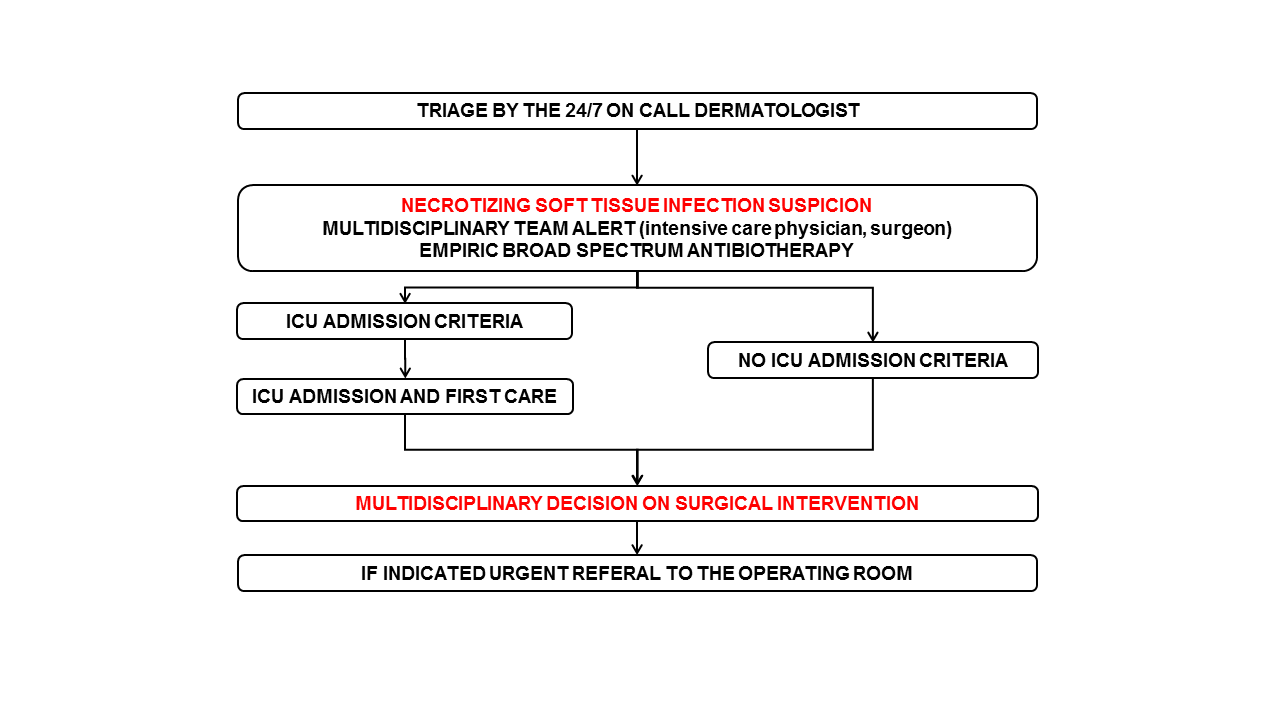


**Figure S3**. **Study flow-chart with excluded patients.**

**
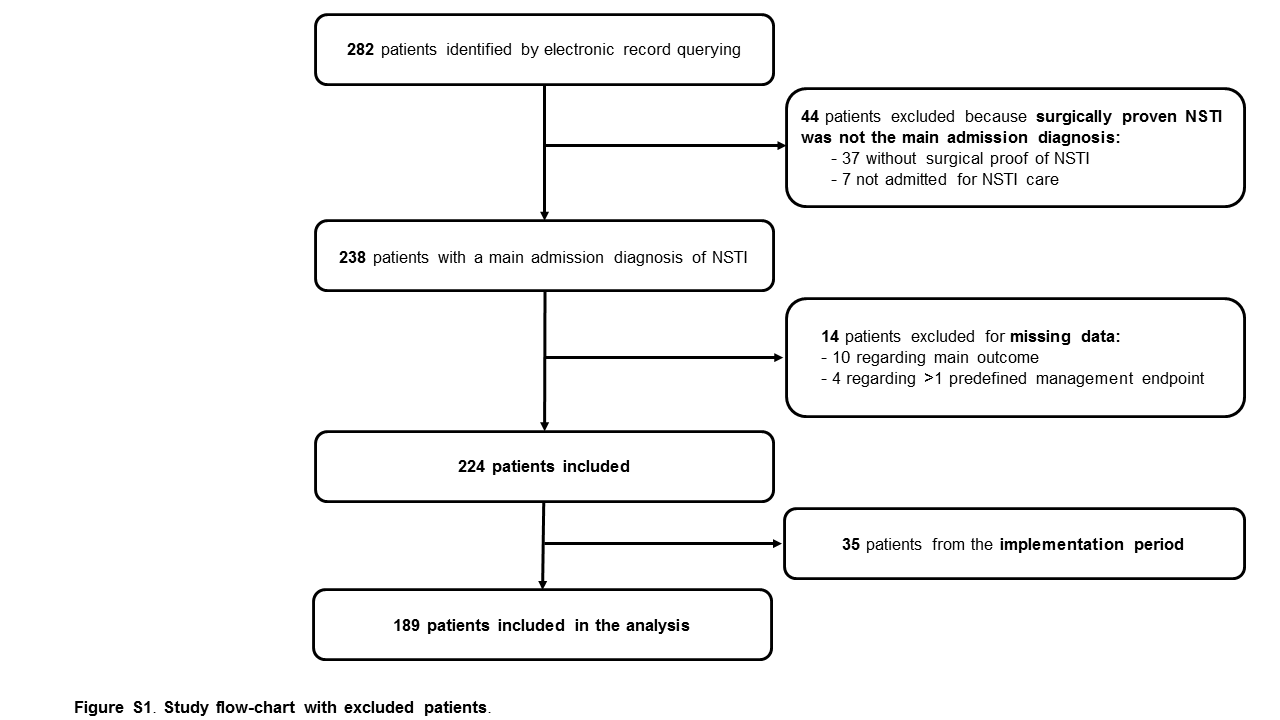
**
