# Supplementary material for: Impact of a multidisciplinary care bundle for necrotizing skin and soft tissue infections: a retrospective cohort study
Source: Ann Intensive Care. 2019 Oct 24;9:123. doi: 10.1186/s13613-019-0598-4 (PMC6813408; doi:10.1186/s13613-019-0598-4)
Supplement: Supplementary file 2 — Additional file 2: Appendix S1. Details on the necrotizing soft-tissue infection task force and on bundle items. [file 13613_2019_598_MOESM2_ESM.docx]

**Impact of a multidisciplinary care bundle for necrotizing skin and soft tissue infections: a retrospective cohort study**

**Additional File 2: Appendix S1: Details on the Necrotizing soft-tissue infection task force and on bundle items**

The task force is composed of one or more practitioners from every specialty involved in NSTI management: intensive care medicine, dermatology, infectious diseases, plastic surgery, anesthesia, microbiology and radiology.

The members of the task force meet on a trimesterly basis, reviewing all NSTI cases managed during the past 3 months, establishing correcting measures aimed at improving patient management, and designing and conducting research projects in the field of NSTI.

Concerning the direct translation of the task force to patient management, task force members are always involved at three different time points: referral, admission and follow-up. Patients are evaluated by specialists according to their needs and clinical presentation, as follows: 1) The 24/7 on call dermatologist, intensive care practitioner or plastic surgeon may be contacted by doctors outside the institution for referral of a patient with suspected NSTI. In case the patient is admitted, all of these specialists are informed; 2) Upon hospital admission, patient evaluation is always conducted by an available task force member or another practitioner from their ward, and is always multidisciplinary, involving at least a dermatologist and a plastic surgeon and, according to clinical severity, an intensivist; 3) The decision for urgent surgical exploration and debridement is made and the patient is transferred to the operating room as soon as possible; 4) Infectious disease specialists, microbiologists and radiologists are alerted by these first members if needed. Microbiologists advise on the choice of microbiological investigations, including biomolecular or metagenomic methods, and implement these if necessary, to guide antibiotic therapy in accordance with infectious disease specialists.

During follow-up, multidisciplinary bedside reevaluation is encouraged 24 h after the first surgery and later on if needed. The aim is to define a common management strategy with clear reevaluation criteria and time points regarding both surgical and medical treatment.

Some items from the bundle deserve to be described in more detail.

Operating room access was facilitated through several measures. First, there was an important communication process with all practitioners involved (emergency room doctors, dermatologists, surgeons, anesthesiologist, intensive care practitioners) regarding diagnosis criteria and the importance of early surgery. This is crucial, especially in less experienced centers to try to decrease both time to diagnosis and time to surgical decision. Second, NSTI was inscribed in the list of “Surgical Emergencies” of the anesthesiology department. This list encompasses the surgical procedures listed as urgent that must overcome programmed interventions for operating room access. This was a major issue regarding the logistics once the surgical decision was made (stretcher-bearers, operating room cleaning, on call anesthesiologists and OR nurses). Of course, time to diagnosis remains an important issue, but time to surgical decision and to OR access seem unacceptable delays that we sought to reduce. Of note, delay to surgery also includes time needed for early management of eventual organ failures before first surgery.

Local guidelines for empiric antibiotherapy included an intravenous broad-spectrum beta-lactam (Piperacillin-Tazobactam 4 g/6h in prolonged perfusion for abdominoperineal infections or Amoxicillin-clavulanate 100 mg/kg/24h in 3 daily doses) with addition of Clindamycin 600 mg/6h in case of limb NSTI. Aminoglycosides were added only in case of shock and Carbapenems or Vancomycin were only used in case of identified risk factors for ESBL or MRSA infections. These guidelines were based on most recent international and French guidelines as well as our local ecology.

Regarding specimen collection, the bundle recommended obtaining -as soon as the diagnosis was suspected- a set of blood cultures and performing subcutaneous or bullae puncture on the affected area. During surgery multiple biopsies were recommended, at least one from a necrotic area and one from viable tissue for microbiological analysis.
